# Symptoms, impacts, and suitability of the Pulmonary Arterial Hypertension – Symptoms and Impact (PAH-SYMPACT™) questionnaire in patients with chronic thromboembolic pulmonary hypertension (CTEPH): a qualitative interview study

**DOI:** 10.1186/s41687-021-00327-9

**Published:** 2021-06-29

**Authors:** Brooke Currie, Evan Davies, Amélie Beaudet, Larissa Stassek, Leah Kleinman

**Affiliations:** 1 Evidera Inc., Bethesda, MD USA; 2 Actelion Pharmaceuticals Ltd., Allschwil, Switzerland; 3 Evidera Inc., Seattle, WA USA

**Keywords:** CTEPH, PAH-SYMPACT™, Qualitative study, Patient-reported outcome, Health-related quality of life

## Abstract

**Background:**

Chronic thromboembolic pulmonary hypertension (CTEPH) is a rare form of pulmonary hypertension caused by blood clots and scar tissue in the blood vessels of the lungs. Health-related quality of life is often significantly impaired in patients with CTEPH. However, a better understanding of how CTEPH symptoms affect patients’ lives is needed to optimally assess the impact of the disease and treatment.

**Objectives:**

This qualitative study aimed to better understand the symptoms of CTEPH and how they affect patients’ lives, as well as to determine the appropriateness of the Pulmonary Arterial Hypertension – Symptoms and Impact (PAH-SYMPACT™) questionnaire for use in this patient population.

**Methods:**

Adults diagnosed with CTEPH, recruited from two clinical sites in the US, participated in one-to-one qualitative telephone interviews. They described their experience of CTEPH symptoms and the impact these symptoms have on their lives. They also provided feedback on the comprehensibility and relevance of the PAH-SYMPACT™‘s instructions, items, and response options.

**Results:**

Participants (*N* = 12) had a mean age of 62.5 years. Two thirds were female and most (83%) had undergone pulmonary endarterectomy and/or balloon pulmonary angioplasty. The most frequently endorsed symptoms were shortness of breath (endorsed by all 12 participants), fatigue (11 participants), and lightheadedness (10 participants). All participants identified shortness of breath as an “extremely important” symptom, and seven participants rated fatigue as “extremely important.” The most frequent impacts of CTEPH were on ability to walk quickly (endorsed by all 12 participants), ability to walk up inclines or stairs (11 participants), and ability to carry things (11 participants). The PAH-SYMPACT™ items were relevant to most participants and reflected their experience of CTEPH. All participants indicated that no important CTEPH symptoms were missing from the PAH-SYMPACT™. Overall, the instructions, items, and response options of the PAH-SYMPACT™ were clear and easy to understand.

**Conclusions:**

The symptoms and impacts experienced by patients with CTEPH align with items included in the PAH-SYMPACT™. The PAH-SYMPACT™ appears to be fit for purpose for assessing disease status in patients with CTEPH.

**Supplementary Information:**

The online version contains supplementary material available at 10.1186/s41687-021-00327-9.

## Background

Chronic thromboembolic pulmonary hypertension (CTEPH) is a form of pulmonary hypertension (PH) characterized by small vessel arteriopathy and fibro-thrombotic obstructions of large pulmonary arteries [1, 2]. A recent systematic literature review of CTEPH epidemiology suggested an incidence in adults of 3.1 to 6.0 per million and a prevalence of 25.8 to 38.4 per million [3]. In a prospective, large-scale, international European registry, 3-year survival was estimated at 89% in patients with newly diagnosed CTEPH who underwent pulmonary endarterectomy (PEA) surgery and 70% in those who did not [1].

Patients with CTEPH have poor health-related quality of life (HRQoL). In a cross-sectional analysis using data for patients enrolled in the Pulmonary Hypertension Association Registry, HRQoL was significantly worse in patients with CTEPH than in those with idiopathic pulmonary arterial hypertension (PAH), another form of PH with a different etiology than CTEPH [4, 5]. Patient-reported outcome (PRO) instruments are useful in evaluating CTEPH symptoms and their impact on patients’ daily lives and general wellbeing [6]. However, the limited number of clinical trials on CTEPH have not generally assessed HRQoL as an outcome, so there is little data pertaining to these outcomes in patients with CTEPH [6, 7]. Those studies that have assessed HRQoL in patients with CTEPH have used PRO instruments that are either generic (e.g. Short Form-36 Health Survey [SF-36], EQ-5D) or that were designed to evaluate PH, respiratory disease, or heart failure [6, 7]. There is a lack of evidence for the sensitivity of these instruments to adequately capture CTEPH-specific outcomes.

To the authors’ knowledge, the only published PRO instruments for PH that assess both symptoms and impacts and whose validation work included patients with CTEPH are the Cambridge Pulmonary Hypertension Outcome Review (CAMPHOR) [8] and the emPHasis-10 [9]. The CAMPHOR assesses symptoms, functioning, and HRQoL, and has been used to measure the symptoms and impacts of CTEPH [7]. However, the instrument includes 65 items [8, 10], which could make it challenging to administer in both real-world clinical practice and clinical trials. Moreover, because of its limited response options, which are a combination of dichotomous responses and 3-point Likert scales, the CAMPHOR may lack adequate sensitivity to properly capture patient experiences [8]. The emPHasis-10 is a simple 10-item instrument developed to assess HRQoL in patients with PH during routine visits to the clinic [9]. It focuses on a narrow set of PH-related impacts and does not comprehensively capture the symptoms or impacts attributed to PH. Moreover, only 16% of patients included in the primary validation study for the emPHasis-10 had CTEPH [9]. Furthermore, neither the CAMPHOR nor the emPHasis-10 was developed according to US Food and Drug Administration (FDA) guidance for PRO measures [11]. Several other symptom-specific instruments such as Mahler’s dyspnea index [12, 13] have been used in patients with CTEPH, but their scope is limited to a single symptom concept. Alternative, more comprehensive instruments for assessing both symptoms and impacts in CTEPH patients are therefore needed.

The Pulmonary Arterial Hypertension – Symptoms and Impact (PAH-SYMPACT™) was developed according to US FDA guidance for PRO measures to evaluate the symptoms and impacts of PAH [11, 14]. The psychometric properties of the PAH-SYMPACT™ have been evaluated using data from the phase IIIb SYMPHONY trial of the endothelin receptor antagonist macitentan in patients with PAH. These validation analyses supported the PAH-SYMPACT™ as a reliable instrument for assessing symptoms of PAH and their impact on patients [10]. Specifically, the PAH-SYMPACT™ demonstrated high internal consistency reliability and test-retest reliability, was able to differentiate between patients based on their disease severity and was sensitive to improvements in disease severity.

Patients with CTEPH exhibit symptoms similar to those in PAH, including shortness of breath, fatigue, and lightheadedness [10, 15]. However, the general lack of qualitative research in CTEPH has resulted in a limited understanding of how CTEPH symptoms affect patients’ lives. The objectives of the current study were to better understand CTEPH symptoms and their impact on patients’ lives, as well as to assess the content validity of the PAH-SYMPACT™ for use in patients with CTEPH.

## Methods

### Study design

This was a cross-sectional, observational study conducted between December 2019 and April 2020. Adult patients diagnosed with CTEPH were recruited from two clinical sites in the US to participate in a qualitative one-to-one telephone interview. For both clinical sites, central ethics approval (Ethical and Independent Review Services: 18158) and local ethics approval (Colorado Multiple Institutional Review Board: 18–2587; Cleveland Clinic Institutional Review Board: 19–1069) was obtained before any participant recruitment procedures were initiated. All participants provided written informed consent for participation and permission for the telephone interview to be audio-recorded and transcribed. The study was conducted in accordance with the International Council for Harmonisation guideline for Good Clinical Practice and the Health Insurance Portability and Accountability Act.

### Participants

Eligible participants were English-speaking adults aged 18–85 years diagnosed with inoperable CTEPH or persistent/recurrent CTEPH after balloon pulmonary angioplasty (BPA) or persistent/recurrent PH after PEA (including PEA followed by BPA). Potential participants were excluded if they had a pre-existing cardiovascular condition that would confound discussion of CTEPH symptoms and their impact or any clinically relevant medical condition that the investigator judged would interfere with consent or the study procedures.

### PAH-SYMPACT™

The PAH-SYMPACT™ is a 23-item PRO instrument for assessing symptoms and impacts of PAH [10, 14]. It consists of 11 symptom items with a 24-h recall period, 11 impact items with a 7-day recall period, and one item on oxygen use in the previous 24 h. The symptom items are divided into cardiopulmonary and cardiovascular domains, and the impact items are divided into physical and emotional/cognitive domains. Symptom and impact domain scores (range 0 to 4) are calculated as the sum of the scores for the items included in the domain divided by the number of items in the domain. Mean weekly symptom domain scores are calculated across the days in a given week; a minimum of four out of seven days of symptom data are needed in order to calculate a weekly score. For all domains, a higher score indicates more severe symptoms/impacts. For this study, references to “PAH” were removed from the PAH-SYMPACT™‘s title and instructions to avoid confusing the study participants, who did not have PAH. No references to CTEPH were added to the PAH-SYMPACT™.

### Qualitative interviews

The telephone interviews were conducted by two health outcomes research professionals (LS and MW), each with a master’s degree. The study sites provided the interviewers with contact information for patients who were eligible and willing to participate and who consented to be contacted for this study. Potential participants were then contacted by one of the researchers (MW) by telephone to schedule the interviews.

The interviews were conducted using a semi-structured qualitative interview guide (Supplementary Table 1), developed based on the study objectives and the research team’s experience of conducting qualitative interviews. At the beginning of the interview, the researchers introduced themselves by name to participants, and provided their employer’s name (Evidera) and the reason for doing the interviews. Written informed consent was then obtained.

The interviews included concept elicitation, followed by completion of the PAH-SYMPACT™ and cognitive debriefing. Concept elicitation included open-ended questions to elicit participants’ experiences of CTEPH symptoms and the impacts they have on their lives. Participants were asked during the interview to rate the importance of each symptom and impact they endorsed on a 4-point numeric rating scale ranging from 0 (not important) to 3 (extremely important). They were also asked to select the symptom or symptoms that were “most bothersome or severe” and the impact or impacts that were “most difficult to cope with.”

After concept elicitation, participants completed the PAH-SYMPACT™ before proceeding to cognitive debriefing, where they were asked for their feedback on the comprehensibility of the PAH-SYMPACT™‘s instructions and the relevance and clarity of the items and response options. Participants were also asked to indicate the minimum score change they would consider meaningful for the symptom and impact domains and for specific symptom items (shortness of breath, fatigue, cough, and lightheadedness) and impact items (walking uphill, carrying things, and feeling worried).

### Analyses

Interviews were audio-recorded and the audio files were then transcribed verbatim by a third-party vendor. The transcripts were reviewed (by MW) to remove any identifiable information about participants and to correct any obvious transcription errors. The final “cleaned” transcripts were not returned to participants for comment or correction.

Participant responses from the concept elicitation and cognitive debriefing parts of the interview were analyzed using ATLAS.ti version 8.3 (ATLAS.ti Scientific Software Development GmbH, Berlin, Germany) [16]. Transcripts were coded using a coding framework developed by one of the researchers (LS) based on the main symptoms and impacts included in the interview guide and the specific goals of the interviews. The coding framework was subsequently revised based on review by a third researcher (BC) and findings from the initial interviews. Two researchers (LS and MW) independently coded one transcript using the coding framework. The dual-coded transcript was reviewed to confirm that the researchers had captured all relevant responses and were interpreting and using codes consistently and as intended. One of the researchers (MW) then coded the remaining transcripts and the other researcher (LS) checked the quality of the coded transcripts.

All quantitative data were entered into DataFax, an optical character recognition software package that is compliant with Part 11 of Title 21 of the US FDA Code of Federal Regulations (DF/Net Research, Inc., Seattle, WA). Descriptive statistics were calculated using SAS 9.4 (SAS Institute Inc., Cary, NC).

For the concept elicitation data, saturation of concepts was evaluated after 12 patients had been interviewed. A saturation grid was developed by arranging the transcripts chronologically and dividing them into four groups of three transcripts. The grid was used to determine whether the inclusion of additional study participants would capture any new concepts [17].

## Results

### Participants and sociodemographic and clinical characteristics

Twelve participants (mean [SD] age 62.5 [15.2] years) were enrolled and completed a telephone interview. Interviews lasted an average of 68 min (standard deviation [SD] 9.9 min). Most of the participants were female (*n* = 8) and white (*n* = 7) (Table 1). Seventy-five percent of participants had at least some college education. Mean (SD) time since diagnosis of CTEPH was 4.2 (3.7) years, and half of the participants had a World Health Organization (WHO) functional class of II, indicating slight limitation of physical activity [18]. Most participants (*n* = 10) had undergone PEA and/or BPA. Their current treatments included anticoagulants (*n* = 12), diuretics (*n* = 10), soluble guanylyl cyclase stimulators (*n* = 7), and supplemental oxygen (*n* = 7).

**Table 1:** Participant sociodemographic and clinical characteristics

Characteristic	*N* = 12
Age (years), mean (SD)	62.5 (15.2)
Gender, n	
Male	4 (33)
Female	8 (67)
Ethnicity, n	
Hispanic or Latino	2 (17)
Not Hispanic or Latino	8 (67)
No response	2 (17)
Racial background^a^, n	
Black or African American	4 (33)
White	7 (58)
Other (participant specified “Hispanic”)	1 (8)
Marital status, n	
Single	1 (8)
Married	7 (58)
Divorced	3 (25)
Widowed	1 (8)
Employment status, n	
Employed, full-time	2 (17)
Retired	8 (67)
Disabled	2 (17)
Highest level of education, n	
Secondary/high school	3 (25)
Some college	5 (42)
College degree	4 (33)
Time since diagnosis of CTEPH (years)^b^, mean (SD)	4.2 (3.7)
Most recent WHO functional class^b^, n	
I	3 (25)
II	6 (50)
III	3 (25)
Current treatment^a,b^, n	
Oxygen	7 (58)
Diuretics	10 (83)
Anticoagulants	12 (100)
Soluble guanylyl cyclase stimulator	7 (58)
Endothelin receptor antagonists	5 (42)
Fibrinogen	1 (8)
Phosphodiesterase type 5 inhibitors	4 (33)
Prostacyclin/prostacyclin analogues/prostacyclin receptor agonists	3 (25)
Intervention^a,b^, n	
BPA only	3 (25)
PEA surgery only	6 (50)
BPA and PEA surgery	1 (8)
Neither BPA nor PEA surgery	2 (17)
Comorbid conditions^a,b^, n	
None	1 (8)
Anxiety	2 (17)
Chronic obstructive pulmonary disease	2 (17)
Depression	2 (17)
Diabetes with chronic complications	4 (33)
Hypertension	3 (25)
Obstructive sleep apnea	3 (25)
Rheumatological disease	1 (8)
Other health condition(s)^c^	11 (92)

*BPA* Balloon pulmonary angioplasty, *CTEPH* Chronic thromboembolic pulmonary hypertension, *PEA* Pulmonary endarterectomy, *SD* Standard deviation, *WHO* World Health Organization

^a^Responses were not mutually exclusive

^b^Information was captured by the clinical sites using a case report form

^c^Other health conditions, each reported by *n* = 1 participant unless otherwise specified, were splenectomy (*n* = 2), chronic anemia, idiopathic pulmonary arterial hypertension, coronary artery disease, connective tissue disease, transient ischemic attack, thyroid dysfunction, obesity, osteoarthritis, prostate cancer, renal insufficiency, scoliosis, paralyzed hemidiaphragm (right), and spherocytosis

### Concept elicitation

#### Saturation

Saturation of symptom and impact concepts was achieved within the first six interviews. Fourteen symptom concepts were raised by the first group of three participants and five new symptom concepts (chest pain, fainting, body pain or ache, balance issues, and dizziness) were collectively endorsed by the second group of three participants (Supplementary Table 2). Twenty impact concepts were endorsed by the first group of three participants and two new impact concepts (dependence on others and isolation) were endorsed by the second group of three participants (Supplementary Table 3). Aside from two symptom concepts that were judged to overlap with previously endorsed concepts, no new symptom or impact concepts emerged from the third or fourth groups of three participants. No further patients were therefore recruited.

#### Symptom concepts

Symptoms endorsed by participants are summarized in Table 2, and representative participant quotations are included in Supplementary Table 4. The most frequently endorsed symptoms were shortness of breath, fatigue, and lightheadedness. Shortness of breath was spontaneously reported by all 12 participants, who described the sensation of shortness of breath as “*scary*” (participant ID: 001–006) or feeling like “*breathing through a straw*” (001–001), “*gasping for air*” (001–002), or “*drowning*” (002–010). Fatigue was reported by 11 participants, many of whom described it as feeling “*tired*” (*n* = 7) or as “*exhaustion*”/feeling “*exhausted*” (*n* = 7)*.* Individual participants described fatigue as “*bone crushing*” (002–003) or “*like narcolepsy*” (002–010), or reported feeling so tired that they would need to sleep (during the daytime) for “*a couple hours”* to feel *“rejuvenate*[d]” (002–002). Lightheadedness was reported by 10 participants, three of whom reported occasionally fainting or passing out. Four participants reported that lightheadedness occurred when they stood up or bent over too quickly. Others experienced it while “*talking for prolonged periods of time*” (002–003) or when they “*went up a flight of steps*” (002–002).

**Table 2:** Summary of endorsed symptom concepts

Concept	Endorsement, *n* (%)		
	Spontaneous	Through probing	Total
Shortness of breath	12 (100)	0	12 (100)
Fatigue	6 (50)	5 (42)	11 (92)
Lightheadedness	3 (25)	7 (58)	10 (83)
Rapid heartbeat	4 (33)	5 (42)	9 (75)
Lack of energy	2 (17)	7 (58)	9 (75)
Swelling in ankles or legs	3 (35)	4 (33)	7 (58)
Swelling in stomach area	2 (17)	4 (33)	6 (50)
Cough	3 (25)	3 (25)	6 (50)
Heart palpitations	0	5 (42)	5 (42)
Chest pain	2 (17)	3 (25)	5 (42)
Chest tightness	2 (17)	2 (17)	4 (33)
Fainting	3 (25)	0	3 (25)
Body pain or ache	2 (17)	0	2 (17)
Coughing up blood	2 (17)	0	2 (17)
Headache	1 (8)	0	1 (8)
Balance issues	1 (8)	0	1 (8)
Dizziness	1 (8)	0	1 (8)
Legs feeling heavy	1 (8)	0	1 (8)
Purple lips	1 (8)	0	1 (8)

Other frequently reported symptoms included rapid heartbeat, which was reported by nine participants, with participants describing it as “*pounding*” and “*racing*” (001–005) or “*tachycardia*” (001–001). Nine participants reported lack of energy. Individual participants described the lack of energy as “*physical weakness*” (001–004) or feeling “*worn out*” (002–007), “*your muscles feel*[ing] *like Jell-O*” (001–004), or *“not so much* [feeling] *tired as I just don’t feel like I have the energy”* (001–003). One participant considered that lack of energy was similar to fatigue, whereas other participants indicated that they were different, noting that lack of energy is not feeling like doing anything whereas fatigue is more a feeling of being tired. Swelling in the ankles or legs was reported by seven participants. Individual participants described their legs as feeling “*very heavy and tight”* (001–001) or making it necessary to “*walk really slow*” (002–010).

Most participants who reported the above symptoms indicated that symptom severity would vary with level of exertion or activity. The only exception was lack of energy, where only two participants (22%) indicated that it varied with activity.

When asked to rate the importance of individual symptoms on a scale from 0 (“not at all important”) to 3 (“extremely important”), all 12 participants rated shortness of breath as “extremely important” and seven participants rated fatigue as “extremely important” (mean importance rating 2.55). Other symptoms with a mean rating of 2 (“somewhat important”) or higher included lack of energy (2.44), swelling in the ankles or legs (2.14), and rapid heartbeat (2.00). Symptoms identified as the “most bothersome or severe” were shortness of breath (*n* = 8), fatigue (*n* = 4), and lack of energy (*n* = 2).

#### Impact concepts

Endorsed impacts are summarized in Table 3 and representative participant quotations are included in Supplementary Table 5. The most frequently endorsed impacts were impaired ability to walk, carry things, and perform housework or chores.

**Table 3:** Summary of endorsed impact concepts

	Endorsement, *n* (%)		
Item	Spontaneous	Through probing	Total
Ability to walk in general	10 (83)	2 (17)	12 (100)
Walking quickly	3 (25)	9 (75)	12 (100)
Walking slowly	3 (25)	7 (58)	10 (83)
Walking on flat surfaces	4 (33)	6 (50)	10 (83)
Walking up inclines/stairs	8 (67)	3 (25)	11 (92)
Carrying things	2 (17)	9 (75)	11 (92)
Housework or chores	7 (58)	3 (25)	10 (83)
Hobbies or social activities	9 (75)	0 (0)	9 (75)
Feeling frustrated or angry	1 (8)	8 (67)	9 (75)
Feeling worried or anxious	3 (25)	5 (42)	8 (67)
Feeling sad or depression	3 (25)	3 (25)	6 (50)
Dependence on others	0	6 (50)	6 (50)
Dealing with oxygen	5 (42)	0	5 (42)
Mental functioning	2 (17)	3 (25)	5 (42)
Self-care activities	3 (25)	1 (8)	4 (33)
Work	4 (33)	0	4 (33)
Talking	3 (25)	0	3 (25)
Standing	2 (17)	0	2 (17)
Financial impacts	2 (17)	0	2 (17)
Clothing and shoe fit problems *(due to swelling)*	2 (17)	0	2 (17)
Feeling embarrassed	1 (8)	0	1 (8)
Isolation	1 (8)	0	1 (8)

All 12 participants reported that their general ability to walk was negatively affected, and most participants (*n* = 10) clearly attributed this to their shortness of breath. Individual participants found walking difficult because they had a “*hard time breathing*” (001–001) or were “*breathing pretty heavily*” (001–007). Most participants (*n* = 8) even had difficulty breathing while walking short distances. One participant described difficulty walking “*five feet*” (002–008), and another had difficulty walking “*to the bathroom*” (001–005). Most or all participants indicated that their walking ability was impacted when walking quickly (*n* = 12) or slowly (*n* = 10) on flat surfaces, or when walking up inclines or stairs (*n* = 11).

Most participants (*n* = 11) reported difficulty with carrying things, which they attributed to shortness of breath or fatigue. The difficulty they experienced ranged from being unable to carry a “*10-pound bag*” to the car (001–005) to struggling to carry their purse (001–004, 001–006, 002–010).

Ten participants indicated that CTEPH affected their ability to do housework or chores and attributed this to shortness of breath, fatigue, lack of energy, or lightheadedness. Activities with which participants reported having difficulty included “*doing laundry*” (002–003), performing “*roof work*” (001–005), and “*changing lightbulbs in the ceiling*” (001–004).

Most participants (*n* = 9) also reported difficulties with participating in hobbies or social activities. They described difficulties running or jogging (*n* = 3), riding a bike (*n* = 2), or playing sports like basketball or golf (*n* = 1). Participants also reported problems socializing with friends (*n* = 4) and effects on family time (*n* = 5), which manifested as being unable to “*go to the grandkids’ football games and baseball games”* (002–008) or “*go out with friends anymore”* (001–006). Three participants reported that they were no longer able to ride a motorcycle, travel, or dance (*n* = 1 each).

Nine participants reported that CTEPH caused them to feel frustrated or angry, and eight reported feeling worried or anxious. Reasons for their frustration or anger included inability to realize their “*dream about traveling*” after retiring (002–003) and CTEPH making them feel like they were no longer “normal” (002–010). Reasons why participants felt worried or anxious included concerns about their health or their future (*n* = 2) and fear of blood clots forming while flying (*n* = 1).

Among impacts endorsed by at least half of participants, hobbies or social activities was scored as 3 (“extremely important”) by five of the six participants who rated it (mean importance rating 2.83). Walking activities (2.64–2.80) and carrying things (2.73) also had high mean importance ratings. Other impacts with a mean importance rating of 2 (“somewhat important”) or more included housework or chores (2.40), feeling frustrated or angry (2.25), and feeling worried or anxious (2.25). No clear trend was observed for the impacts participants felt were “most difficult to cope with.”

### Responses to the PAH-SYMPACT™

As indicated by their responses to the PAH-SYMPACT™, most participants (*n* = 8) had used oxygen in the previous 24 h (Table 4). Each of the 11 PAH-SYMPACT™ symptom items had been experienced by at least two participants in the previous 24 h, and each of the 11 PAH-SYMPACT™ impact items had been experienced by at least three participants in the previous 7 days.

**Table 4:** PAH-SYMPACT™ responses

	Response, n (%)				
In the past 24 h …Did you use oxygen?	No	Yes			
	4 (33)	8 (67)			
In the past 24 h …	0 (no symptom at all)	1 (mild)	2 (moderate)	3 (severe)	4 (very severe)
“How would you rate your shortness of breath?”	2 (17)	3 (25)	7 (58)	0	0
“How would you rate your fatigue?”	3 (25)	1 (8)	7 (58)	1 (8)	0
“How would you rate your lack of energy?”	3 (25)	5 (42)	4 (33)	0	0
“How would you rate the swelling in your ankles or legs?”	5 (42)	6 (50)	1 (8)	0	0
“How would you rate the swelling in your stomach area?”	7 (58)	2 (17)	1 (8)	2 (17)	0
“How would you rate your cough?”	10 (83)	0	2 (17)	0	0
“How would you rate your heart palpitations (heart fluttering)?”	9 (75)	1 (8)	2 (17)	0	0
“In the past 24 h … How would you rate your rapid heartbeat?”	7 (58)	1 (8)	4 (33)	0	0
“How would you rate your chest pain?”	10 (83)	2 (17)	0	0	0
“How would you rate your chest tightness?”	8 (67)	3 (25)	1 (8)	0	0
“How would you rate your lightheadedness?”	4 (33)	7 (58)	1 (8)	0	0
In the previous 7 days …	Yes, with no difficulty at all	Yes, with a little difficulty	Yes, with some difficulty	Yes, with much difficulty	No, not able at all
“Were you able to walk slowly on a flat surface?”	6 (50)	4 (33)	2 (17)	0	0
“Were you able to walk quickly on a flat surface?”	3 (25)	3 (25)	2 (17)	2 (17)	2 (17)
“Were you able to walk uphill?”	2 (17)	2 (17)	3 (25)	5 (42)	0
“Were you able to carry things?”	3 (25)	2 (17)	3 (25)	4 (33)	0
“Were you able to do light indoor household chores?”	6 (50)	3 (25)	2 (17)	1 (8)	0
“Were you able to wash or dress yourself?”	9 (75)	2 (17)	0	1 (8)	0
In the previous 7 days …	Not at all	A little bit	Some	Quite a bit	Very Much
“How much did you need help from others?”	5 (42)	3 (25)	3 (25)	1 (8)	0
In the previous 7 days …	Yes, with no difficulty at all	Yes, with a little difficulty	Yes, with some difficulty	Yes, with much difficulty	No, not able at all
“Were you able to think clearly?”	8 (67)	2 (17)	2 (17)	0	0
In the previous 7 days …	Not at all	A little bit	Somewhat	Very	Extremely
“How sad did you feel?”	7 (58)	2 (17)	1 (8)	2 (17)	0
“How worried did you feel?”	5 (42)	4 (33)	1 (8)	2 (17)	0
“How frustrated did you feel?”	4 (33)	4 (33)	3 (25)	0	1 (8)

*PAH-SYMPACT™,* Pulmonary Arterial Hypertension – Symptoms and Impact

A score of 0 in the previous 24 h (symptoms items) or in the previous 7 days (impact items)

A score of 1 or more in the previous 24 h (symptoms items) or in the previous 7 days (impact items)

### Cognitive debriefing

#### Relevance of the PAH-SYMPACT™ items

When asked about the symptom items collectively, half of participants (*n* = 6) indicated that all 11 symptom items were relevant to their experiences of CTEPH, even if they had not reported experiencing a given symptom in the previous 24 h. Four participants indicated that cough was not relevant to them. The other symptom items were each relevant to at least 10 participants. Some participants acknowledged that, even if they had not experienced a particular symptom, the symptom could still be relevant to others with CTEPH.

When asked about the impact items collectively, all 12 participants indicated that all 11 impact items were relevant to their experiences of CTEPH, even if they had not experienced a given impact in the previous 7 days. All participants also indicated that the oxygen use item was relevant.

#### Overall thoughts on the PAH-SYMPACT™

All participants indicated that the instructions were clear. Generally, participants reported no problems in understanding the symptom and impact items, although the meaning of “chest tightness” was unclear to one participant. All participants were able to distinguish between all or most symptom items. However, three participants felt that the following pairs of items were similar: lack of energy and fatigue, heart palpitations and rapid heartbeat, and chest pain and chest tightness (*n* = 1 each).

When asked, all participants reported that no CTEPH-related symptoms were missing from the PAH-SYMPACT™. However, some participants indicated that the PAH-SYMPACT™ failed to capture all impacts of CTEPH. One participant suggested that the impact on general day-to-day activities could also be considered (without specifying which activities), as well as feelings of isolation, anger, and panic. Another participant spontaneously commented that difficulty climbing stairs could be added.

All participants reported that the response options were clear for all PAH-SYMPACT™ items. However, four participants indicated that it would have been useful to be able to indicate whether factors such as comorbid health conditions or stressful life situations also contributed to their responses. One participant expressed a problem with recall for doing light indoor household chores and washing and dressing oneself. The same participant suggested that more detail or clarity for the needing help from others item would help them to distinguish between the help they felt they needed and the help they actually received.

### Meaningful change

**Fig. 1:**
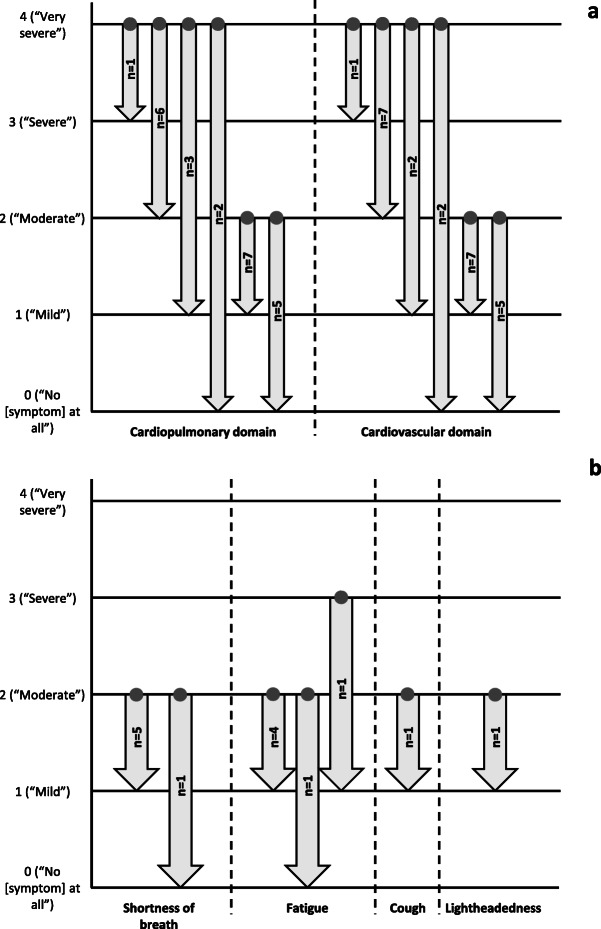
Meaningful change for symptoms. **a** Symptom domains. **b** Individual symptom items. The starting point when discussing meaningful change (denoted by the dots at the base of the arrows) was a hypothetical severity level of 4 (“Very severe”) or 2 (“Moderate”) for the symptom domains; for the symptom items, the starting point was the response given when completing the PAH-SYMPACT™. For each symptom item, only participants who scored the item as 2 or higher when completing the PAH-SYMPACT™ were included in the meaningful change discussions. Cardiopulmonary domain: shortness of breath, fatigue, lack of energy, swelling in the ankles or legs, swelling in the stomach area, and cough; cardiovascular domain: heart palpitations, rapid heartbeat, chest pain, chest tightness, and lightheadedness

For the symptom and impact domains, as well as for specific symptom and impact items, participants were asked to consider the score they gave when responding to the PAH-SYMPACT™ and to indicate the minimum score change they would consider to be meaningful. For both the cardiopulmonary and cardiovascular domains, a one-point score change from 2 (“Moderate”) to 1 (“Mild”) would be considered meaningful by most participants (Fig. 1a). When starting from a score of 4 (“Very severe”), only one participant indicated that a one-point improvement for both domains would be meaningful. Most participants who participated in meaningful change discussions for shortness of breath and fatigue indicated that a one-point score change from 2 (“Moderate”) to 1 (“Mild”) would be meaningful (Fig. 1b).

**Fig. 2:**
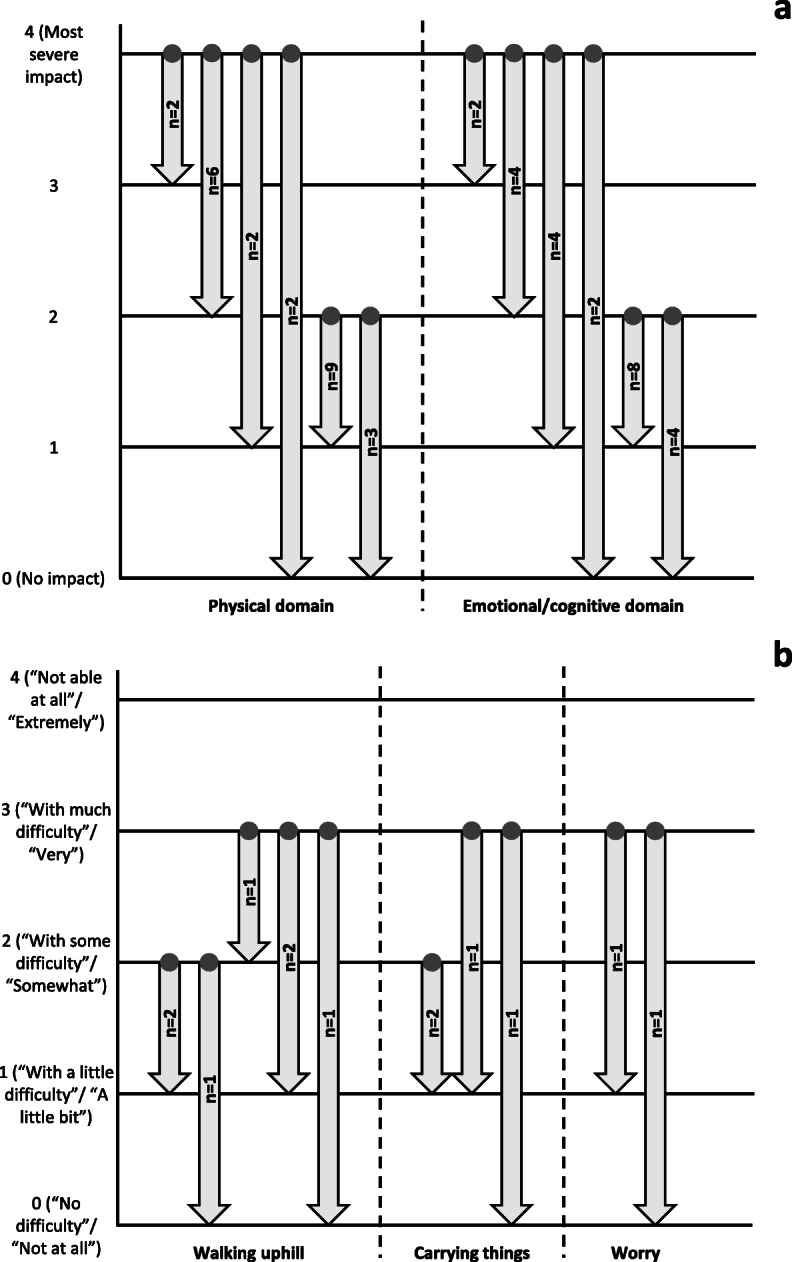
Meaningful change for impacts. **a** Impact domains. **b** Individual impact items. The starting point when discussing meaningful change (denoted by the dots at the base of the arrows) was a hypothetical severity level of 4 or 2 for the impact domains; for the impact items, the starting point was the response given when completing the PAH-SYMPACT™. For each impact item, only participants who scored the item as 2 or higher when completing the PAH-SYMPACT™ were included in the meaningful change discussions. Physical domain: walking slowly on a flat surface, walking quickly on a flat surface, walking uphill, carrying things, doing light indoor household chores, washing or dressing oneself, and needing help from others; emotional/cognitive domain: thinking clearly, feeling sad, feeling worried, and feeling frustrated

For the physical and emotional/cognitive domains, most participants would consider a one-point score change from 2 to 1 to be meaningful; two participants indicated that a one-point change from 4 to 3 would be meaningful (Fig. 2a). Participants who participated in meaningful change discussions for walking uphill and carrying things identified various different score changes as meaningful (Fig. 2b).

## Discussion

In this qualitative interview study, all PAH-SYMPACT™ symptom and impact items were relevant to most or all participants and their experiences of CTEPH. Shortness of breath was rated as “extremely important” by all participants and as the “most bothersome or severe” symptom by most participants. CTEPH symptoms clearly affected participants’ ability to engage in certain activities. Notably, ability to walk was affected in all participants. This study adds to what is a limited amount of literature on how patients with CTEPH experience their disease and increases understanding of how their HRQoL is affected.

The PAH-SYMPACT™ fulfils the need for a content valid instrument for assessing symptoms and impacts in patients with CTEPH. The instructions, items, and response options of the PAH-SYMPACT™ were generally clear to all participants in the present study. Although participants mentioned that no important symptom items were missing, minor additions or changes to the impact items were suggested. However, few of the suggestions were made by more than one participant. Four participants felt that the contribution of comorbid conditions and other factors to their responses should be captured, but this is not feasible for a disease-specific questionnaire. Some insight into the possible influence of these other factors can instead be obtained through other means, for example by examining medical records/clinical data in the case of comorbid conditions. For these reasons, no changes to the PAH-SYMPACT™ are recommended, and the questionnaire appears to be fit for purpose for assessing disease status in patients with CTEPH.

Participants would generally consider a one-point improvement as meaningful when starting from a score representing moderate symptoms or impacts; when starting from a score representing more severe symptoms or impacts, they would generally consider a two-point or greater score change as meaningful. Thus, the more severe a symptom or impact, the greater an improvement may need to be for it to be considered meaningful. However, establishing usable meaningful change or responder thresholds will require a larger quantitative study.

Limitations of the present study include limited generalizability of the results because of the small sample (*N* = 12), the high average level of education (nine participants had at least some college education), and recruitment of participants by only two sites in a single country. Also, most participants had undergone PEA or BPA, which may have resulted in symptom improvements and a decreased impact due to CTEPH. This may explain why proportions of participants reporting certain symptoms and impacts were higher for the concept elicitation portion of the interview (where participants reported on their lifetime experience of symptoms/impacts) compared to the PAH-SYMPACT™ (reported experience in the previous 24 h/7 days). The sample may not have been representative of CTEPH patients who have not undergone surgical intervention, many of whom suffer from severe symptoms. Although the sample included two patients with inoperable CTEPH, this number was too small for a meaningful comparison with patients who had undergone PEA or BPA. In order to capture a sample that is more diverse in terms of ethnicity, education level, and CTEPH severity, future studies should include definitive demographic targets and work with clinical sites that have access to broad patient populations. Finally, because 11 of the 12 interview transcripts were only coded by a single coder, the inherent biases of the coder may have affected how the data was captured and conceptualized. However, the coding was comprehensively checked by a second coder.

This study is the first step in the process of validating the PAH-SYMPACT™ in patients with CTEPH. Although the PAH-SYMPACT™ has been psychometrically evaluated in patients with PAH [10], it is important to assess its performance and measurement properties in CTEPH patients to confirm it is fit for use in this population. Additional analyses using blinded data from a phase 3 trial are planned. These analyses will include an investigation of the PAH-SYMPACT™ domain structure and scoring by confirmatory factor analysis; evaluation of internal consistency reliability, test-retest reliability, construct validity, and responsiveness; and estimation of meaningful within-patient change thresholds.

## Conclusions

This qualitative study demonstrated that the symptoms and impacts experienced by patients with CTEPH align with items included in the PAH-SYMPACT™, and that the PAH-SYMPACT™ was well understood by the study sample. The results support the content validity of the PAH-SYMPACT™ for use in patients with CTEPH.

## Supplementary Information

**Additional file 1: Supplementary Table 1**. Outline of the semi-structured qualitative interview guide. **Supplementary Table 2**. Concept saturation grid for symptoms. **Supplementary Table 3**. Concept saturation grid for impacts. **Supplementary Table 4**. Representative quotations for the most frequently endorsed symptom concepts. **Supplementary Table 5**. Representative quotations for the most frequently endorsed impact concepts.

## Data Availability

The data sharing policy of Janssen Pharmaceutical Companies of Johnson & Johnson is available at https://www.janssen.com/clinical-trials/transparency. As noted on this site, requests for access to the study data can be submitted through the Yale Open Data Access (YODA) Project site at http://yoda.yale.edu.

## Abbreviations

BPA: Balloon pulmonary angioplasty
CTEPH: Chronic thromboembolic pulmonary hypertension
FDA: Food and Drug Administration
HRQoL: Health-related quality of life
PAH-SYMPACT™: Pulmonary Arterial Hypertension – Symptoms and Impact
PEA: Pulmonary endarterectomy
PH: Pulmonary hypertension
PRO: Patient-reported outcome
SD: Standard deviation
SF-36: Short Form-36 Health Survey
US: United States
WHO: World Health Organization

## References

[1] Delcroix M, Lang I, Pepke-Zaba J, Jansa P, D’Armini AM, Snijder R, Bresser P, Torbicki A, Mellemkjaer S, Lewczuk J, Simkova I, Barberà JA, de Perrot M, Hoeper MM, Gaine S, Speich R, Gomez-Sanchez MA, Kovacs G, Jaïs X, Ambroz D, Treacy C, Morsolini M, Jenkins D, Lindner J, Dartevelle P, Mayer E, Simonneau G (2016). Long-term outcome of patients with chronic thromboembolic pulmonary hypertension: Results from an international prospective registry. Circulation.

[2] Wilkens, H., Lang, I., Behr, J., Berghaus, T., Grohe, C., Guth, S., … Mayer, E. (2011). Chronic thromboembolic pulmonary hypertension (CTEPH): Updated recommendations of the Cologne Consensus Conference 2011. *International Journal of Cardiology*, *154*(Suppl 1), S54–S60. 10.1016/S0167-5273(11)70493-4. 10.1016/S0167-5273(11)70493-4 22221974

[3] Leber L, Beaudet A, Muller A (2021). Epidemiology of pulmonary arterial hypertension (PAH) and chronic thromboembolic pulmonary hypertension (CTEPH): Identification of the most accurate estimates from a systematic literature review. Pulmonary Circulation.

[4] Narasimmal, S. P., Pugliese, S., Bull, T. M., De Marco, T., McConnell, J. W., Lammi, M. R. ... Al-Naamani, N. (2020). Quality of life of patients with chronic thromboembolic pulmonary hypertension (CTEPH) and idiopathic pulmonary arterial hypertension (IPAH): The Pulmonary Hypertension Association Registry (PHAR). *American Journal of Respiratory and Critical Care Medicine*, *201*, A6048.

[5] Humbert M (2010). Pulmonary arterial hypertension and chronic thromboembolic pulmonary hypertension: Pathophysiology. European Respiratory Review.

[6] Mathai SC, Ghofrani HA, Mayer E, Pepke-Zaba J, Nikkho S, Simonneau G (2016). Quality of life in patients with chronic thromboembolic pulmonary hypertension. The European Respiratory Journal.

[7] Newnham M, Bunclark K, Abraham N, Ali S, Amaral-Almeida L, Cannon JE, Doughty N, Ng C, Ponnaberanam A, Sheares K, Speed N, Taboada D, Toshner M, Tsui S, Jenkins DP, Pepke-Zaba J (2020). CAMPHOR score: Patient-reported outcomes are improved by pulmonary endarterectomy in chronic thromboembolic pulmonary hypertension (CTEPH). The European Respiratory Journal.

[8] McKenna, S. P., Doughty, N., Meads, D. M., Doward, L. C., & Pepke-Zaba, J. (2006). The Cambridge Pulmonary Hypertension Outcome Review (CAMPHOR): A measure of health-related quality of life and quality of life for patients with pulmonary hypertension. *Quality of Life Research*, *15*(1), 103–115. 10.1007/s11136-005-3513-4. 10.1007/s11136-005-3513-4 16411035

[9] Yorke J, Corris P, Gaine S, Gibbs JSR, Kiely DG, Harries C, Pollock V, Armstrong I (2014). emPHasis-10: Development of a health-related quality of life measure in pulmonary hypertension. The European Respiratory Journal.

[10] Chin, K. M., Gomberg-Maitland, M., Channick, R. N., Cuttica, M. J., Fischer, A., Frantz, R. P., … Badesch, D. B. (2018). Psychometric validation of the Pulmonary Arterial Hypertension-Symptoms and Impact (PAH-SYMPACT) questionnaire: Results of the SYMPHONY trial. *Chest*, *154*(4), 848–861. 10.1016/j.chest.2018.04.027. 10.1016/j.chest.2018.04.027 29705220

[11] US Food & Drug Administration. (2009). Guidance for industry. Patient-reported outcome measures: Use in medical product development to support labeling claims. Available from: https://www.fda.gov/media/77832/download. [cited 26 August 2020] 10.1186/1477-7525-4-79 PMC1629006 17034633

[12] Mahler DA, Weinberg DH, Wells CK, Feinstein AR (1984). The measurement of dyspnea. Contents, interobserver agreement, and physiologic correlates of two new clinical indexes. Chest.

[13] Witek TJ, Mahler DA (2003). Minimal important difference of the transition dyspnoea index in a multinational clinical trial. The European Respiratory Journal.

[14] McCollister, D., Shaffer, S., Badesch, D. B., Filusch, A., Hunsche, E., Schüler, R., … IRB information for the 5 clinical sites (2016). Development of the Pulmonary Arterial Hypertension-Symptoms and Impact (PAH-SYMPACT®) questionnaire: A new patient-reported outcome instrument for PAH. *Respiratory Research*, *17*(1), 72. 10.1186/s12931-016-0388-6. 10.1186/s12931-016-0388-6 PMC4908719 27301413

[15] Winter MP, Schernthaner GH, Lang IM (2017). Chronic complications of venous thromboembolism. Journal of Thrombosis and Haemostasis.

[16] Friese, S., & Ringmayr, T. G. (2013). ATLAS.ti 7 user guide and reference. Available from: https://atlasti.com/wp-content/uploads/2014/05/atlasti_v7_manual_201312.pdf?q=/uploads/media/atlasti_v7_manual_201312.pdf. [cited 25 August 2020]

[17] Leidy NK, Vernon M (2008). Perspectives on patient-reported outcomes: Content validity and qualitative research in a changing clinical trial environment. Pharmacoeconomics.

[18] Vachiery JL, Simonneau G (2010). Management of severe pulmonary arterial hypertension. European Respiratory Review.

