# Supplementary material for: Symptoms, impacts, and suitability of the Pulmonary Arterial Hypertension – Symptoms and Impact (PAH-SYMPACT™) questionnaire in patients with chronic thromboembolic pulmonary hypertension (CTEPH): a qualitative interview study
Source: J Patient Rep Outcomes. 2021 Jun 29;5:51. doi: 10.1186/s41687-021-00327-9 (PMC8241969; doi:10.1186/s41687-021-00327-9)
Supplement: Supplementary file 1 — Additional file 1: Supplementary Table 1. Outline of the semi-structured qualitative interview guide. Supplementary Table 2. Concept saturation grid for symptoms. Supplementary Table 3. Concept saturation grid for impacts. Supplementary Table 4. Representative quotations for the most frequently endorsed symptom concepts. Supplementary Table 5. Representative quotations for the most frequently endorsed impact concepts. [file 41687_2021_327_MOESM1_ESM.docx]

**Supplementary Table 1** Outline of the semi-structured qualitative interview guide

| **Themes to be explored** | **Examples of questions** |
| --- | --- |
| **Concept Elicitation** | |
| CTEPH Diagnosis | How long ago were you diagnosed?  What sort of issues or problems were you experiencing that you led you to the doctors in the first place? |
| CTEPH Symptoms | What types of symptoms have you experienced as a result of CTEPH?  **For each symptom reported:** terms used to describe it, description of the severity, variability (e.g., based on exertion or time of day) |
| CTEPH Symptom Relevance/ Importance Rating | How important or relevant is **[*symptom*]** to your experience of CTEPH? In other words, when you think of your overall experience with CTEPH, how much does **[*symptom*]** play a role in that experience? Rating scale: “Not at all important” (0), “A little important” (1), “Somewhat important” (2), or “Extremely important” (3) |
| CTEPH Symptom Bother | Which symptom(s) is most bothersome or severe to you and why? |
| Impacts of CTEPH symptoms on functioning and quality of life | Do your CTEPH symptoms impact your life on a regular basis? If so, how? What specific activity/activities are affected by your CTEPH symptoms? |
| CTEPH Impact Relevance/ Importance Rating | How important or relevant is **[*impact*]** to your experience of CTEPH? In other words, when you think of your overall experience with CTEPH, how much does **[*impact*]** play a role in that experience? Rating scale: “Not at all important” (0), “A little important” (1), “Somewhat important” (2), or “Extremely important” (3) |
| CTEPH Impact Coping | Which impact(s) is/are the most difficult for you to cope or deal with? |
| **Cognitive Debriefing of the PAH-SYMPACT** | |
| Symptom item relevance and missing concepts | Did each of these questions apply to your current experiences with CTEPH?  Are there any additional CTEPH symptoms that you experience but were not mentioned in these 12 questions? |
| Overall appropriateness of phrasing of the symptom items | What did you think of the way the questions were phrased?  Would you recommend that any of the questions be worded differently? |
| Clarity of the symptom item instructions | In your own words, what are these instructions telling you to do?  Is there a better way that you would suggest wording these instructions to make them more clear or easier to understand? |
| Appropriateness of the symptom item administration schedule | This part of the questionnaire (the first 12 questions about symptoms) has been designed to be completed every day at the same time for a week. Do you think that people with CTEPH would be able to do that? |
| Relevance of the oxygen item | Do you ever use supplemental oxygen? What answer did you choose and why? |
| Relevance and clarity of individual symptom items | **For each of the 11 symptom items:** What answer did you choose and why? What does **[symptom]** mean to you? |
| Meaningful change for a subset of symptom items | **For select symptom items:** If you start taking a treatment and then are asked to answer this question again, what is the minimum amount of change you would need to see in your answer to feel like that treatment had given you a meaningful benefit? |
| Clarity of the response options | Are the answer choices clear in meaning and easy to understand?  What do each of the answer choices mean to you? |
| Meaningful change for the two symptom domains | **For each of the domains:** The researchers take the average of your score on those **[domain items]** so the average is from 0 (no symptoms) to 4 (very severe symptoms), so the lower the score the less severe your symptoms are, overall.  If your average score from these symptom items started at a 4 before taking a treatment, which is the worst possible (most severe) score, where would you need to move on the scale to feel you had experienced meaningful improvement in those symptoms? In other words, what is the smallest change you would need to see in your average score to feel like you benefitted from the treatment?  If your average score started at a 2, which would indicate moderate symptoms, where would you need to move on the scale to feel you had experienced meaningful improvement in those symptoms? |
| ***(The same sequence of probing was then repeated for the impact items and impact domains)*** | |

**Supplementary Table 2** Concept saturation grid for symptoms

| Symptom concept | *n* (%) | Transcript group | | | | |
| --- | --- | --- | --- | --- | --- | --- |
|  |  | 1 (*n* = 3) | 2 (*n* = 3) | 3 (*n* = 3) | 4 (*n* = 3) |  |
| Shortness of breath | 12 (100) | ✓ | – | – | – |  |
| Fatigue | 11 (92) | ✓ | – | – | – |  |
| Lightheadedness | 10 (83) | ✓ | – | – | – |  |
| Lack of energy | 9 (75) | ✓ | – | – | – |  |
| Rapid heartbeat | 9 (75) | ✓ | – | – | – |  |
| Swelling in ankles or legs | 7 (58) | ✓ | – | – | – |  |
| Swelling in stomach area | 6 (50) | ✓ | – | – | – |  |
| Cough | 6 (50) | ✓ | – | – | – |  |
| Chest tightness | 5 (42) | ✓ | – | – | – |  |
| Heart palpitations | 5 (42) | ✓ | – | – | – |  |
| Wet-Productive cough | 4 (33) | ✓ | – | – | – |  |
| Dry cough | 3 (25) | ✓ | – | – | – |  |
| Coughing up blood | 2 (17) | ✓ | – | – | – |  |
| Headache | 1 (8) | ✓ | – | – | – |  |
| Chest pain | 5 (42) | – | ✓ | – | – |  |
| Fainting | 3 (25) | – | ✓ | – | – |  |
| Body pain or ache | 2 (17) | – | ✓ | – | – |  |
| Balance issues | 1 (8) | – | ✓ | – | – |  |
| Dizziness | 1 (8) | – | ✓ | – | – |  |
| Legs feeling heavy^a^ | 1 (8) | – | – | – | ✓ |  |
| Purple lips^b^ | 1 (8) | – | – | – | ✓ |  |

Tick marks identify the group of transcripts in which each symptom concept was first raised or endorsed.

^a^Assessed as likely related to swelling in ankles or legs and possibly related to lack of energy

^b^Assessed as related to (and a result of) shortness of breath

**Supplementary Table 3** Concept saturation grid for impacts

| Impact concept | *n* (%) | Transcript group | | | | |
| --- | --- | --- | --- | --- | --- | --- |
|  |  | 1 (*n* = 3) | 2 (*n* = 3) | 3 (*n* = 3) | 4 (*n* = 3) |  |
| Ability to walk | 12 (100) | ✓ | – | – | – |  |
| Walking quickly impacted | 12 (100) | ✓ | – | – | – |  |
| Walking uphill or climbing stairs | 11 (92) | ✓ | – | – | – |  |
| Carrying things | 11 (92) | ✓ | – | – | – |  |
| Walking slowly impacted | 10 (83) | ✓ | – | – | – |  |
| Walking on flat surfaces | 10 (83) | ✓ | – | – | – |  |
| Housework or chores | 10 (83) | ✓ | – | – | – |  |
| Hobbies or social activities | 9 (75) | ✓ | – | – | – |  |
| Feeling frustrated or angry | 9 (75) | ✓ | – | – | – |  |
| Feeling worried or anxious | 8 (67) | ✓ | – | – | – |  |
| Feeling sad or depression | 6 (50) | ✓ | – | – | – |  |
| Dealing with oxygen | 5 (42) | ✓ | – | – | – |  |
| Mental functioning | 5 (42) | ✓ | – | – | – |  |
| Self-care activities | 4 (33) | ✓ | – | – | – |  |
| Work school volunteering | 4 (33) | ✓ | – | – | – |  |
| Talking | 3 (25) | ✓ | – | – | – |  |
| Standing | 2 (17) | ✓ | – | – | – |  |
| Financial impacts | 2 (17) | ✓ | – | – | – |  |
| Clothing and shoe fit problems^a^ | 2 (17) | ✓ | – | – | – |  |
| Feeling embarrassed | 1 (8) | ✓ | – | – | – |  |
| Dependence on others | 6 (50) | – | ✓ | – | – |  |
| Isolation | 1 (8) | – | ✓ | – | – |  |

Tick marks identify the group of transcripts in which each impact concept was first raised or endorsed.

^a^Due to swelling

**Supplementary Table 4** Representative quotations for the most frequently endorsed symptom concepts

| Symptom concept | Quotation [Participant ID] |
| --- | --- |
| Shortness of breath | “*…a feeling of breathing through a straw […]. I would be going through the motions […] cannot feel like I can breathe quick enough or get enough air in. By the time I’m finished exhaling I feel like I already need to be taking my next breath to try to feel like I’ve caught my breath…*” [001-001] |
|  | “*Well, you can’t breathe, you’re gasping for air. When they first found out that I had it, I felt—it happened in the middle of the night—like somebody hit me with a baseball bat on my side.*” [001-002] |
|  | “*It’s that feeling that you’re suffocating and you can’t breathe—I mean it's so scary when you can’t breathe.*” [001-006] |
|  | *“Every time I’ve lost my breath, I felt like I was drowning, like this was it. There was no other breath to be had…It’s awful*.” [002-010] |
|  | “*…mostly I’m good. Any time you exert yourself to do anything, depending on what you’re doing, [the shortness of breath] gets worse*.” [001-003] |
|  | *“Anytime that I’m pushing myself a little further than I should, I may get a little breathless or feel a little weak... Lifting something heavy will make me breathless and stepping out in cold air.”* [001-004] |
|  | *“[The shortness of breath] was pretty severe. Small tasks […] get my breathing relatively heavy. And the recovery time from that would be longer…So, […], it would be difficult to like jog or anything like that, or going up a flight of stairs would’ve been something that I’ll be recovering from for quite some time.”* [001-007] |
| Fatigue | “*The fatigue, maybe just if I was doing household chores or cutting grass, I would have to come in and basically sit down and take a nap. That was how fatigued I was and tired, and would sleep for a couple hours and rejuvenate…At the time I was still working, and after a day of work I would be totally exhausted*.” [002-002] |
|  | *“I mean, when I used to talk about the fatigue, I used to call it bone crushing. […]. I mean, there was no comparison. There’s almost no way of explaining it…And if I would just walk in the door and plop down on the bed, not necessarily to take a nap but just to lay down.”* [002-003] |
|  | “*[The severity of the fatigue is] definitely activity related, because I don’t just get tired in general, it’s only when I exert*.” [001‑003] |
|  | *“…Initially, when I was diagnosed I had noticed it would be harder to work out or harder to get through a 12-hour shift at work and still feel that I could do anything else that day pretty acutely over the year or two before my diagnosis. Things were rapidly declining with my functional status prior to surgery. […] I could not or did not have any energy to do anything else or household tasks. I think I’ve improved; I am able to work full-time again and I also workout multiple days of the week,...”* [001‑001] |
| Lightheadedness | “*I would be doing something and just get – and I’d be on oxygen…I would get lightheaded. Not dizzy. […]. I would get lightheadedness doing certain things. Talking for prolonged periods of time I can get lightheaded*. […].” [002-003] |
|  | “*It was pretty bad. If I went up a flight of steps, I would get dizzy, have to stop and let my body recover before I would proceed*.” [002-002] |
|  | “*Sometimes it's—you know, the room is spinning. Sometimes it's just a little bit of that, where I’ll get ready to stand up and go or I’ll move a few steps and feel lightheaded*.” [001-006] |
| Rapid heartbeat | “*Rapid heartbeat—I would not say palpitations, because I’ve never had any indications of having Afib…but definitely tachycardia even still now post-surgery*.” [001-001] |
|  | “*So, any exertion my heartbeat will increase very rapidly…I would not be able to exert myself maybe more so than just walking on the treadmill at a slight incline before my heartbeat would be too high that I would need to back off from an activity*.” [001-001] |
|  | *“Sometimes, very rarely…[rapid heartbeat happens when I’m] just sitting still.”* [002-010] |
|  | “*It changes all during the day. Like, I’m sitting right now and there’s no problem at all, and then it may, this afternoon, I may be sitting here and all of a sudden, I might feel—I’ll feel like my heart is racing. I call it pounding*…” [001-005] |
| Lack of energy | *“[…] it’s just like I just don’t feel like I feel like doing anything. […] I’m not so much tired as I just don’t feel like I have the energy…”* [001-003] |
|  | “*General body weakness, especially upper body weakness. I can’t carry anything with any decent weight, even just carrying like my purse and oxygen becomes difficult*.” [001-004] |
| Swelling in ankles or legs | *“It happens on a daily basis. […], because I can feel my skin having pressure put on it while they’re swelling, and my legs will start to feel just very heavy and tight, and …”* [001-001] |
|  | *“for years, my ankles would swell, and like for my job I traveled a lot. So, every time I flew, […] I would always swell really bad…you’d have to walk from your hotel room to the meeting room, and it just was always difficult for me…I just would have to walk really slow…or I would take a taxi…”* [002-010] |

**Supplementary Table 5** Representative quotations for the most frequently endorsed impact concepts

| Impact concept | Quotation [Participant ID] |
| --- | --- |
| Ability to walk in general | *“…as I’m walking between buildings at work I’ll get a feeling of just my limbs are exponentially heavier and like it’s harder to move my feet... I can move, but I feel like it’s physically difficult to move, because I’m having a hard time breathing or I’m so tired.”* [001-001] |
|  | *“I just couldn’t breathe. I mean, I couldn’t walk five feet.”* [002-008] |
|  | *“Like walking from my job to the parking lot would have been something that would’ve had me breathing pretty heavily…”* [001-007] |
|  | *“I’d be around the house [...], I’d get up and I’d walk to the bathroom, and I’m breathing—I’m starting to breathe hard. And I said this is not right, I shouldn’t be breathing hard just from that short walk, ...”* [001-005] |
| Carrying things | *“[I have] general body weakness, especially upper body weakness. I can’t carry anything with any decent weight, even just carrying like my purse and oxygen becomes difficult.”* [001-004] |
|  | “*Carrying the groceries into the house is not too bad…If those are real light bags…but if I have to pick up something heavy…for instance, if I go to the hardware store and I grab a 10-pound bag of deicer and try to walk that out to the car, I don’t feel like I could make it. […].”* [001-005] |
|  | *“…if I carried more than my weight and the oxygen portal, I couldn’t carry anything else. I went to not carrying a purse…I couldn’t do it. It was very hard.”* [001-006] |
|  | *“My husband carries my purse wherever we go. So, yeah, I can’t carry anything. It just makes it harder and then I can’t walk as far either.”* [002-010] |
| Housework or chores | *“Just doing laundry. I can no longer bend down and pick anything up off the floor. […]…just clearing out a dishwasher, putting dishes away, is so difficult.”* [002-003] |
|  | *“I know I can’t do a lot of the things that I used to do around my house. I won’t do any roof work, I won’t climb a ladder…I’m sure that has impacted my work around the house, I always have to have someone come in and do certain things for me…”* [001-005] |
|  | *“…like changing lightbulbs in the ceiling, I mean, that’s just not something I have any business doing, so, yeah, I have somebody do that. […]. Even heavy gardening stuff, if it involves like serious digging, I have to have someone come and do that for me.”* [001-004] |
| Hobbies or social activities | *“I can’t do some of the things I used to do, like running distances and riding a bike, it has impact as far as that, and everyday things that I would normally do. I can’t do the things I used to do, or I would have to slow down and do it in portions.”* [002‑002] |
|  | *“I had to, you know, not be able to go to the grandkids’ football games and baseball games because I couldn’t walk that far.”* [002-008] |
|  | *“I couldn’t go out with friends anymore. I couldn’t see my granddaughter.”* [001-006] |
| Feeling frustrated or angry | *“Just frustrated that I’m not normal. Frustrated that I have to wear this pump, the medicines. I hate that. […]. I got to take my walker and my wheelchair, my scooter, my medicine. So, it’s all just very frustrating.”* [002-010] |
|  | *“you dream about retirement and you dream about traveling when you retire…And the loss of your expectations that’s one of the things that I get angry about, I guess.”* [002-003] |
|  | *“I definitely felt frustrated prior to diagnosis, because of the nonspecific nature of the symptoms, and I felt that it was very hard to be taken seriously when I had been having a decline in my exercise capacity and my feeling of well-being for a couple of years…[…].”* [001-001] |
| Feeling worried or anxious | *“there is definitely anxieties of uncertainty for the future of how we would maintain a livelihood […]…even just if I’m walking somewhere, and we’re outside and I see a hill coming up, […].”* [001-001] |
|  | *“Putting it into words it’s very, very difficult for what CTEPH patients go through, like fear, constant fear that more blood clots are forming or can form at any time. So, if you should decide to fly, is that going to cause – and even though you get up and you walk every hour like you’re supposed to do and you wear the compression stockings, you still have that fear.”* [002-003] |
